# Eating Behaviors of Children with Autism—Pilot Study

**DOI:** 10.3390/nu13082687

**Published:** 2021-08-03

**Authors:** Anna Brzóska, Beata Kazek, Karolina Kozioł, Agnieszka Kapinos-Gorczyca, Małgorzata Ferlewicz, Agnieszka Babraj, Anna Makosz-Raczek, Wirginia Likus, Justyna Paprocka, Paweł Matusik, Ewa Emich-Widera

**Affiliations:** 1 Child Development Support Center “Persevere”, Kępowa 56, 40-583 Katowice, Poland; annabrzoska@poczta.onet.pl (A.B.); beakazek@op.pl (B.K.); malgorzata.m.ferlewicz@gmail.com (M.F.); agabialowas@wp.pl (A.B.); a.makoszraczek@gmail.com (A.M.-R.); 2 Department of Pediatrics and Pediatric Endocrinology, Faculty of Medical Sciences, Medical University of Silesia, 40-583 Katowice, Poland; koziolkarola@gmail.com (K.K.); marekwidera@wp.pl (E.E.-W.); 3 Department of Pediatric Neurology, Faculty of Medical Sciences, Medical University of Silesia, 40-583 Katowice, Poland; endocrin@wp.pl; 4 CZP Feniks, Daily Ward for Children and Adolescents, Młyńska 8, 44-100 Gliwice, Poland; aga27.11@interia.eu; 5 Department of Anatomy, School of Health Sciences, Medical University of Silesia, 40-752 Katowice, Poland; wirginia.likus@gmail.com

**Keywords:** autism spectrum disorder, breastfeeding, complementary food, screen time

## Abstract

Autism Spectrum Disorder (ASD) is the most recognized neuropsychiatric disorder of childhood. Comorbid conditions (such as feeding disorders) are more common among people with autism than among the general population. The most frequent somatic disorders in autistic children include the gastrointestinal disorders observed in 46–91% of patients. The purpose of this study was the evaluation of the nutrition of children with autism, with particular emphasis placed on feeding in the first year of life, in comparison to the group of healthy peers. Participants included 75 Caucasian children (41 children diagnosed with pure autism, and the control group consisting of 34 children without autistic traits). The analysis was performed based on a questionnaire of own design with the first part devoted to the eating practices of the early infancy. Results: Autistic children, as compared to the healthy peers, presented a shortened time of breastfeeding (the children fell asleep at the breast) (*p* = 0.04), a delayed introduction of dairy products (*p* = 0.001), the need of more trials to introduce new foods (*p* = 0.006), a delayed introduction of foods with solid and lumpy structure (*p* = 0.004), a longer duration of bottle feeding (*p* = 0.005), delayed attempts to eating using own hands (*p* = 0.006) and needed a greater support of parents to divert their attention from food during eating (*p* = 0.05). Conclusions: 1. The dietary problems are more common among children with the autism spectrum disorder than among the population of healthy children, during the first year of life from the time of introducing the complementary foods. 2. The autistic children experience difficulties with eating and require their parents’ additional involvement significantly more often than their healthy peers.

## 1. Introduction

Autism spectrum disorder (ASD) is a neurodevelopmental disorder. The social communication deficit and peculiar behavior are key components in diagnosing ASD (ICD-10; DSM 5). The prevalence of ASD is increasing—the Centers for Disease Control and Prevention (CDCP) reported the prevalence of 1 per 100 in 2006, and 1 per 54 in 2016 [1,2,3].

The etiology of autism remains unknown, but the genetic factors along with the triggering environmental factors as well as epigenetic factors are considered to play the main role [3,4]. Among other tests, ADOS-2 test is regarded as the gold standard diagnostic tool used to assess autism spectrum disorder.

Patients with autism experience many co-occurring health problems. The most frequent somatic disorders in autistic children include the gastrointestinal disorders observed in 46–91% of patients [5,6,7,8]. Similar disorders occur significantly less frequently (6–50%) among children without the autistic disorder [6,7,8,9,10].

Approximately 70–80% of children with ASD (average value, depending on the available literature: 44–99%) are suffering with the sensory processing disorder, which is also affecting their social communication and behavior, while the sensory processing disorder is observed among approximately 5–23% of the general population [11,12]. The dietary habits are related to sensory viewing of the world, as well as they are immediately related to the motor skills, maintaining posture and praxis performance [13,14,15].

Different studies have shown that at least 70–90% of parents of the autistic children report eating and feeding issues (such as difficult eating behaviors, food refusal, food dislikes, food selectivity or limited food repertoire, pica, obsessive eating patterns) with varying degrees of severity, while such difficulties occur in approximately 10–45% of children of the general population (with a higher severity in children aged less than 3 years) [5,7,13,16,17,18].

Eating and drinking (next to breathing) are among the primary functions in the human development. The primary functions develop based on the primitive reflexes. The process of eating involves the mouth and face area and is based on the oral praxis and then pharyngeal and esophageal phase of swallowing. The proper course of eating also requires a harmonious development of the gross motor skills and the manual dexterity, as well as the perception and multisensory processing. These mechanisms often fail the people with autism [18].

The period of early childhood is key in nutrition, because a child does not only learn to ingest the fluid and solid food in the first three years of life, but also the nutritional programming or metabolic programming takes place through nutrition. As far as the ontogeny is concerned, especially during the infancy phase, there is a need of increasing the intake of nutrients per the specific body weight as well as an age specific need of introducing different proportions of nutrients. Studies have shown what influence nutrition has on the intellectual potential, physical fitness, as well as what protection it provides against overweight and obesity and other non-communicable diseases such as cardiovascular diseases or cancer [19,20,21].

Breastfeeding is without a doubt considered to be the optimal method of feeding for newborn babies and infants. The mother’s eating patterns also play an important role during this period. Proper nutrition is necessary in maintaining the proper lactation, as well as it affects the mother and the baby’s physical and mental well-being [22].

The recommendations for the breastfeeding mothers include a higher caloric and fluid intake, supplementation of vitamin D, omega 3 fatty acids, as well as calcium, iron and iodine. Introducing supplements or restrictive diets by the mothers without professional supervision may be harmful for the mother as well as the child. According to the nutritional recommendations for healthy infants during their first 6 months of life, children should be breastfed on demand, as this provides the needed energy and nutrients. Only the supplementation of vitamin K and D is recommended. Lactation has a positive effect on the mother, and it protects the child against different health problems. The length of proper breastfeeding period has not been precisely and clearly determined by any scientific society, but it is generally recommended to continue the lactation as long as the mother and the children desire, for a minimum of 24 months, as recommended by the World Health Organization, WHO, and for a minimum of 12 months, as recommended by the American Academy of Pediatrics, AAP. In case breastfeeding is not possible, the infant formula, which is adapted to the child’s needs, may be introduced. The introduction of the complementary foods should take place between the 17th and 26th week of life and in accordance with the child’s developmental milestones of motor and feeding skills [20,23,24,25].

There is a general understanding that there is a strong relation between nutrition and physical and mental development of both healthy and ill children, but the method of feeding the autistic children and its effects is still not fully understood and remains uncertain [23,26,27].

The purpose of this study was to identify the differences in feeding between the autistic children and the children without the autistic traits in their first year of life.

Novelty of the study we have described relates to the use of the questionnaire of our own design which was developed in the result of the cooperation of researchers and practitioners (medicine doctors and therapists) for the purpose of assessing the eating and feeding of infants diagnosed with ASD. The study group was limited to persons with pure ASD (without any comorbid diseases or disorders) which allowed for the exclusion of any coincidence of other health problems. The testing relates to the period prior to the diagnosis which may allow for the determination of an early onset of the behavioral and health symptoms as well as predictors of developmental disorders in the autistic children. According to our knowledge, similar studies, other than the study conducted on seven children with autism who showed growth failure caused by severe feeding problems starting in the first year of life have been published on the feeding problems in early infancy in children, who were later diagnosed with ASD [28].

This research study is part of a research effort where we are concentrating on the analysis of 206 children with “pure autism”. The objective is to determine the endophenotypes, including the ones related to eating and feeding. While designing our own questionnaire (written by the scientists and practitioners working with ASD), we wanted to outline the areas for further research. At this phase of our work, we want to continue the study with an emphasis placed on the aspects of broadening the diet (introduction of complementary foods), the feeding training, and the parents’ techniques used in dealing with the children’s eating and feeding issues. The identification of the significant differences in eating and feeding in the first year of life would serve as the basis for conducting studies among larger groups as well as validating the questionnaire.

## 2. Materials and Methods

The study materials included two groups of children. First group was the study group which consisted of 41 autistic children. The second group was the control group which consisted of 34 healthy children.

### 2.1. The Characteristic of the Studied Group

The study was performed among the autistic Caucasian children who are under the care of the Neurology Outpatient Clinic at John Paul II Upper Silesian Child Health Centre in Katowice, the Neurology Clinic of the Child Development Support Center “Persevere” in Katowice and the Child and Youth Outpatient Mental Health Clinic “NZOZ Feniks” in Katowice. The following inclusion and exclusion criteria have been applied in selecting the participants for the studied group:

Inclusion criteria:

- Informed and voluntary consent of the child’s parent/legal guardian to participate in the study
- Children aged between 2 and 12 years
- Diagnosis made by an ASD psychiatrist (using gold diagnostic test—ADOS-2)

Exclusion criteria:

- Syndromic autism (additionally diagnosed mental disorder, epilepsy, congenital disorder, genetic disorder).
- Mental retardation

### 2.2. The Characteristic of the Control Group

The control group was recruited from the Caucasian children who have been the patients of pediatric clinics in the cities of Tychy and Katowice. The following inclusion and exclusion criteria have been applied in selecting the participants for the control group:

Inclusion criteria:

- Informed and voluntary consent of the child’s parent/legal guardian to participate in the study
- Children aged between 2 and 12 years
- Absence of pervasive developmental disorder (as confirmed by a child and adolescent psychiatrist, child neurologist, psychologist and educational research scientist)
- Absence of any diagnosis of chronic conditions with the potential of affecting the nutritional status (the digestive tract diseases previously diagnosed)

Exclusion criteria:

- Children younger than 2 years and older than 12 years
- Mental retardation

### 2.3. Study Method

The study was performed based on a diagnostic survey. The study was performed between March 2018 and March 2019. A questionnaire of our own design, developed for the purpose of performing a detailed nutritional assessment and dietary analyses for children, was used in this study. The initial part included the personal information about the child’s sex and date of birth, as well as the date of filling out the questionnaire form. The form consisted of two parts, first of which was the main theme of this publication. It comprised 20 questions relating to a child’s nutrition in the first year of life as well as the nutrition of the child’s mother during breastfeeding. The questions related to the feeding methods in the first year of life (breastfeeding, infant formula) and the introduction of the complementary foods, as well as the occurrence of any nutritional difficulties in the time of introducing the changes in feeding. This research study is a pilot study—it’s a part of a research study of 206 children with pure autism versus 100 healthy children. The first 50 patients were chosen and recruited for both groups, and the questionnaires were sent to them by post.

The parents were informed about the voluntary character of participating in the study as well as its anonymity, and that it is intended for the sole purposes of the research study. The paper version of the questionnaire was filled out by the child’s parents, without the presence of the surveyor, and without any time restrictions.

**Figure 1:**
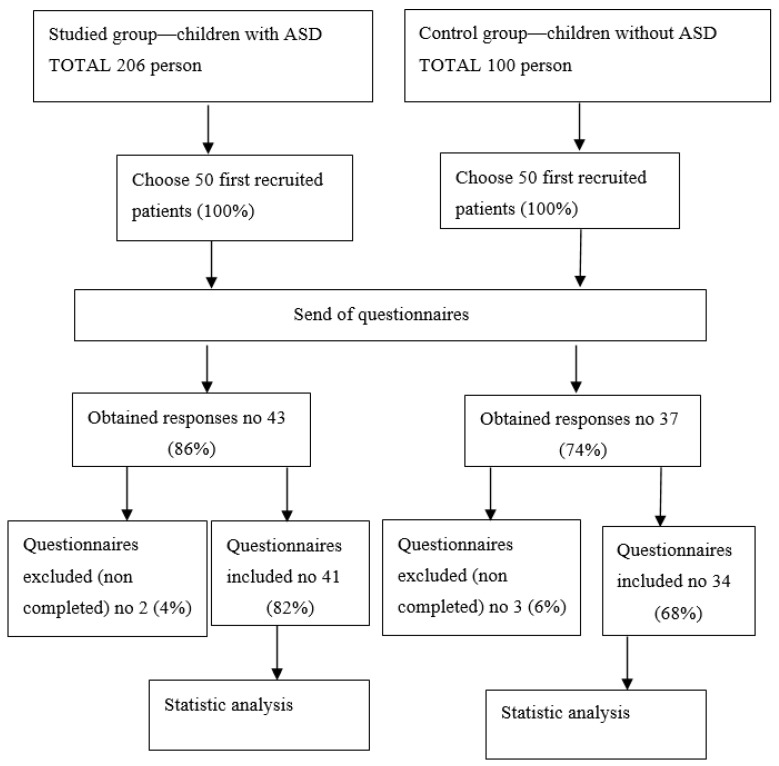
Study flow diagram.

We obtained 43 (86%) questionnaires from the ASD group and 37 (74%) questionnaires from the control group. Some of the questionnaires were not completed in both groups, which resulted in 2 of them being excluded from the ASD group and 3 of them being excluded from the control group. The rest of the questionnaires, i.e., 41 (82%) from the study group and 34 (68%) from the control group, were submitted to the statistical analysis (Figure 1).

### 2.4. Statistical Methods

A number of statistical hypotheses have been verified as part of the statistical analysis of the study results. The Fisher Exact Probability Test was used for the variables in the nominal scale and presented in the four-field tables. The Chi-square Test for Association with Yates continuity correction was used in the multiple-field tables.

While performing the calculations, it was accepted that the negative answers provided for the closed-ended questions were also negative in relation to the variants of the answers proposed in those questions.

For the variables in the ordinal scale (age, grade scale of food/product choice), the nonparametric Mann-Whitney U Test was used in the statistical assessment of the differences in the results of both groups. The test was performed due to the significantly statistically different distributions of the analyzed quantities from the theoretically normal distribution (Shapiro-Wilk Test) (Table 1).

All statistical hypotheses have been verified by applying the standard variable value of 0.05.

## 3. Results

Both groups were homogeneous in terms of sex (*p* = 0.3) (Table 1) and age (*p* = 0.73) (Table 2).

Survey—Part I—Child’s nutrition in the first year of life (20 questions) (see: Supplementary Materials). Based on the data from the survey, it was found that both groups contained children who were breastfed (study group consisting of 37 children vs. control group consisting of 33 children). The comparison of the ages at which the children were when the breastfeeding was ended (calculated in months) did not indicate any statistically significant difference. Similarly, the majority of the breastfeeding mothers in both groups did not use any special diets during breastfeeding. The mothers who provided a positive response to the questions related to the diet of the breastfeeding mother were asked a question regarding the type of the special diet, and there was no difference between the two groups identified as well.

Half of the breastfeeding mothers in both groups reported problems related to breastfeeding—both tests indicate the absence of any statistically significant differences. The analysis of the particular problems (question 3/Table 3), on the other hand, indicated that the significantly more frequent problem reported by the mothers of the autistic children was the shortened time of breastfeeding (the children fell asleep at the breast)—(*p* = 0.04).

In case of the babies fed artificially, the statistical significance was not found for the time of beginning and ending the feeding with the infant formula in comparison with the special infant formula. In both groups, the expanding of diet was begun in the sixth month of the child’s life. Similarly, the result of the test performed to assess the differences in the order in which the complementary foods were introduced did not indicate any statistically significant differences in relation to the introduction of fruits and vegetables. It is worth noting that vegetables were introduced first in both groups (vegetables were introduced as the first complementary food to at least half of the children in each group). The milk products (yogurt, buttermilk) were introduced relatively late; the studied group, in relation to at least half of the children, was placed in the 6th position, and the control group was placed in the 8th position. This is a statistically significant difference (question 7, Table 4; *p* = 0.001).

As far as the other products were concerned (including fruit, meat, fish, chicken eggs), the statistical differences were not found. Significantly more children from the studied group (47.5% vs. 17.7%; *p* = 0.006) required multiple trials of introducing new foods. (Question 8, Table 5).

It was characteristic for the children in the study group to have an approximately 3 times greater need for multiple trials to introduce new foods in comparison to the children in the control group.

The results of the test performed for the assessment of the differences between the groups in relation to the intolerance of particular foods (cow milk, chicken egg, wheat, soy, fish, nuts, chocolate) did not indicate any statistical significance.

Statistically significant difference was found, on the other hand, between the groups in relation to the form of the introduced food for the answer variant “a”: “jars, ready products for infants” (*p* = 0.03) (Question 11, Table 6).

The researchers performed an analysis in relation to the child’s age at which the complementary foods with specific texture were introduced (Table 7, Question 1/10).

The results of this analysis were presented in Table 7. It was found that the children from the studied group were introduced to the foods with lumpy texture (*p* = 0.02) and solid texture (*p* = 0.02) significantly later. Only in case of the foods with fluid or mushy texture, the difference was not found.

A difference was found in answers to the questions related to the preferred texture of food and the intolerance of the particular texture of food by the child older that six months (*p* = 0.004; Question 18, Table 8).

The comparison of the accessories used in feeding the child indicated that a feeding bottle with a nipple was used significantly more often by the children in the studied group (*p* = 0.005), and that these children ate independently using their hands significantly less often (*p* = 0.006) (Question 12, Table 9).

Question 13 was about the need of an additional involvement of the parents or caregivers during the child’s feeding. The children of the studied group required twice as much support from the parents in the form of playing with the child or diverting the child’s attention (*p* = 0.05; Table 10).

It was found that the children from the studied group ate and watched a cartoon at the same time two times more frequently (TV, laptop, tablet, cellular phone). Although the statistical significance was not found for these results, we can indicate certain statistical tendency with such result (*p* = 0.07; Table 11).

A difference between the groups in relation to the analysis of feeding during a sleep or eating together with the other members of the family was not found.

As observed by the caregivers, there was no difference between the groups in relation to the child’s appetite in the first year of life.

The assessment of the differences between the groups in relation to the necessity of introducing the restrictive diet in the child’s first year of life did not indicate any statistical significance.

## 4. Discussion

Children’s eating behaviors are shaped throughout the entire developmental period, beginning from the prenatal period. The genetic predispositions, the family and its eating behaviors, the educational institutions, the peer groups, as well as the mass media affect the child’s eating patterns [29,30].

The analysis of our study conducted on breastfeeding in terms of the length of the breastfeeding period, the diet used by the breastfeeding mother and the occurrence of problems related to lactation indicated that the behaviors of the mothers of the autistic children during the breastfeeding period do not differ significantly from the behaviors of the mothers of the healthy children.

On the other hand, according to the literature, the autistic children are breastfed significantly less often than their healthy peers [31]. The study conducted by M. van’t Hof et al. (2021) on a group of 3,5 thousand mothers with children with ASD indicated that feeding children with formula in infancy was associated with ASD at six years of age [8].

There is a variety of reasons of early cessation of breastfeeding which include somatic, emotional and sensorimotor reasons. Among the sensory processing disorders affecting the breastfeeding process of an infant, the important ones include orofacial tactile hypersensitivity and the tactile hypersensitivity of the entire body, as well as hypersensitivity to smells, tastes and sounds, which are activated during the skin to skin contact, by the mother’s frequent touch, and by the intense taste or smell of breast milk [8]. The autistic children manifest an increased number of many of these hypersensitivities, so we could expect to acquire results similar to those of the other researchers [8,31]. The different result, though, may stem from the differently selected study group, as our studies were conducted on persons with pure autism, which may be related to the lower number of factors contributing to the early termination of breastfeeding.

Most of the mothers of both groups did not use any special diets during the lactation period. The mothers who provided positive answers in this regard indicated mainly the hypoallergenic and dairy-free diets, but these results did not indicate any statistical significance. According to the nutritional recommendations for women during lactation, it is not recommended for the breastfeeding mothers to preventively use any restrictive diets. The classification of the problems related to lactation includes problems related to breastfeeding, such as the incorrect breastfeeding technique, the child’s incorrect behavior (refusing to feed, chaotic feeding), child’s insufficient activity, problems with milk flow, problems with nipples, problems related to the mammary gland, the amount of available milk and the problems related to the state of the child, such as regurgitation, allergies, intolerance or the psychological and emotional problems of the mother. The majority of women in both groups indicated the occurrence of the problems related to lactation. The mothers of the autistic children significantly more often indicated the shortening of the effective feeding time, which was interpreted as the children falling asleep at the breast.

This may indicate, among others, the delayed maturation of the feeding behavior which leads to the shortening of the sucking time. Studies show that the rhythm and the longer sucking sessions are an integral part of the overall development of an organism. It would seem that the developmental abnormalities of the nervous system could be a valuable diagnostic indication. Also, a normal readiness of a newborn/infant for breastfeeding is related to the behavioral organization and energy to act, which is achieved by staying awake, maintaining good posture with the normal muscle tone, and having an interest to suck. It is assumed that there is a relation between the newborn’s method of sucking and the neurodevelopmental and nutritional results later in life [32]. So far, objective knowledge about the measure of endurance, which is a complex matter, is not available. It encompasses the capacity to hold on to the same sucking phase, the duration of the sucking impulse and the sucking amplitude, and/or expression during the sucking session, keeping a constant behavior throughout the sucking session as well as the frequency of breathing and oxygen saturation [32,33,34].

The results of this study prove that the parents of both groups, regardless of the type of milk provided to the children, practiced the feeding on demand method in the first months of the child’s life, which is commonly recommended [23].

The time of introducing the complementary foods is a special time in the child’s first year of life, which optimally should take place between the 17th and the 26th week of life. In this study, the children with the autistic spectrum disorder were introduced to the complementary foods in their 6th month of life, which is recommended and is the same as the time of introducing the complementary foods in the group of the healthy peers [20,23,24,25].

In terms of careful analysis of the order in which the complementary foods were introduced, a few aspects have been highlighted. Vegetables were introducing as the first complementary food to at least half of the children in each group, and fruit was introduced as the second complementary food to the majority of the children, which is considered correct due to the rather bitter taste of vegetables and the sweet taste of fruit. Children with ASD did not differ from the healthy children in terms of the order in which the complementary foods were introduced in their diet, and the broadening of the diet in both groups was implemented in accordance with the recommendations [23,35,36].

The parents of both groups did not indicate any intolerance towards any of the products introduced in the diet within the first 12 months of the child’s life, including the products considered to be allergenic (soy, nuts or chocolate).

This study presents the analysis of the form in which the parents introduced foods different than the mother’s milk or the infant formula. It was statistically confirmed that the parents of the autistic children use the ready, store-bought products designated for feeding infants, commonly referred to as jarred baby food, while the parents of the healthy peers group prepare the food for the children by themselves at home, but this food is prepared solely for the child and for the purpose of feeding it. This provides the children with an opportunity to learn new tastes and fragrances [37,38]. The reluctance of the infants presenting with the autism spectrum disorder or the difficulties with broadening the diet may result from the sensory hypersensitivity and the orofacial motor disorder, secondary to the persistent primary reflex, within this group of patients. Such behaviors may also result from problems with self-regulation [39,40].

The texture of the complementary foods should be adjusted to the child’s developmental skills in terms of eating, such as sucking, swallowing, biting, chewing, as well as small motor skills. In the beginning, the texture should be smooth, then lumpy. In the 9th month, the food should be minced or chopped, and soft parts of food should be placed in the child’s hands. Starting from at least the 12th month of life, the child should eat at the family table, which means no further restrictions of the textures of the products given to the child should be maintained, while of course extra precautions to prevent the child from chocking should be taken. The presented study shows that the autistic children were introduced to the lumpy and solid foods significantly later. The children with ASD were given lumpy foods in the 10th month of life, and the healthy children one month earlier. The solid foods were introduced to the diet of the autistic children after the 12th month of life, which is 2 months later comparing to the healthy children. Our own research indicates that the introduction of the solids foods is performed later than generally recommended.

In this study, attention was also paid to the problems related to preferences or to the opposite—the intolerance and refusal of certain food textures by children in the first year of life and, more specifically, in the time of introducing products different than milk. The children with autism, similarly to the healthy children, did not indicate any preferences/intolerance towards any food texture. The absence of any difference between the groups may be the result of the fact that the food with texture other than mushy was introduced into the diet of the children with ASD, specifically solid food, later in comparison with the healthy children. The period between the 6th and the 10th month of life is strategic in the development of the biting skill by a child, which is only possible through providing the child with foods that have a different texture than fluid or mushy [41].

The diet of the autistic children in the first year of life is less versatile than the diet of the healthy children, and it is based on the ready, store-bought products. Foods, such as the chicken egg yolk, the whole chicken egg, fish or dairy products, as well as foods with lumpy and solid texture, are introduced in their diet at a later time.

It can be concluded that the problems with feeding are more frequent among children with autism, which is consistent with the results of the available studies [16,18]. In the introduction, we have provided the data regarding the percentage of the occurrence of the eating and feeding issues of the autistic children which, according to the available literature, is at least 70–90% as opposed to 10 to 45% in case of the healthy children. The difference between the children of the study and the control groups of the population we are analyzing is not that significant. In our opinion, the difference stems out from the age differences between the groups analyzed by other researchers. The work described in the literature presents older children and a possible participation of children with comorbid health problems (possible genetic disorders or cerebral palsy) and/or development problems (including intellectual disability) in the groups studied by the other researchers. We, on the other hand, present data on the age of infancy. The initial results of the analysis of eating and feeding, conducted among the older children by our team of researchers, shows differences that are more significant between the groups (data so far unpublished).

Nevertheless, based on our analysis, the introduction of the complementary foods in the infants’ diet is significantly more difficult in children with ASD. We have indicated that the children with autism as well as the children without any developmental deficits mostly ate together with the family. Due to that fact, feeding difficulties are challenging for the parents and the rest of the family members significantly increasing the level of stress as well as decreasing the self-assessment of parental competence. Feeding process requires multiple trials and a special involvement of the parent/caregiver in the form of playing with the child or redirecting the child’s attention from the food to the high technology devises (monitors) to make feeding easier and happens more frequently with the children with ASD than with the healthy children. Therefore, the behavioral feeding difficulties are rather frequent in persons with ASD, which is confirmed by the results and is in line with the findings of the researchers [18].

Recent WHO guidelines (2019) advise that children aged under one year old should have no screen time. As it was found, high technology devices—screen time (watching television, playing computer and video games, using mobile phones and tablets)—is associated with the child’s language development. More high technology devices are associated with speech retardation. The likelihood of parents’ use of screens during everyday child routines is also negatively associated with the child’s language development. [42] In addition to that, screen-time (ST) and unhealthy dietary behaviors are closely related starting in the early development age and are mostly passed on from generation to generation [30].

The mealtime in families with autistic children more often requires the use of high technology devices as one of the forms to divert the child’s attention from the food. It is important to teach the parents about the possible negative developmental consequences of such behavior. Based on the presented scientific studies and the experience of the researchers, such conduct may disrupt self-regulation (including the feeling of hunger or satisfaction) and slowdown the development of language and communication skills. In addition to that, unintentional eating may limit the manual abilities and the orofacial motor functions, as well as it may diminish the multisensory perception of the world. This aspect requires profound investigation and analysis to learn about the causality, the possible effects, as well as propose effective solutions to the feeding problems.

The accessories used while feeding infants have also been analyzed in this study. The analysis indicates that a bottle with a nipple is used to feed the children with autism more frequently, and a sippy cup was used to feed the healthy children slightly more often. The children from the control group are given food straight to their hand which promotes a multisensory cognition of food and the shaping of food preferences.

To summarize the analysis of children’s nutrition, the statistical differences in children’s appetite during the newborn and infant period were not found. The mothers of both the autistic and the healthy children describe the children’s appetite in the first year of life as normal.

It is important to realize that a harmonious development and functioning of the central and peripheral nervous systems, including the motor fitness and manual dexterity as well as the visual and auditory perception are the foundations for the development of the communication skills. A dysfunction on any of those levels will result in the psychological, sensory and motor deficits, including speech and communication deficits.

## 5. Limitations of the Study

The presented research is a pilot study presenting preliminary results. The authors used an own questionnaire which has not been validated.The results may be ambiguous due to the retrospective character of the questionnaire and the groups containing a small number of patients. Some of the questions are rather vague which results from the fact that the questionnaire itself is vast and the researchers did not want to overwhelm the parents with too many questions. Nevertheless, this causes the difficulty in interpreting some of the results. Also, a set of many questions does not guarantee too many answers with statistical significance. This shows that caution should be used when formulating conclusions and the study should be continued based on a revised questionnaire. 

## 6. Conclusions

The dietary problems are more common among children with the autism spectrum disorder than among the population of healthy children, during the first year of life from the time of introducing the complementary foods. Further studies are needed in order to identify the exacerbation of these problems as well as to determine the diagnostic and therapeutic procedure to be applied in this group of patients.

The autistic children experience difficulties with eating and require their parents’ additional involvement significantly more often than their healthy peers. The incorrect eating and feeding habits occur more often among the children with ASD than among the healthy children, and that should be the subject of our further, more in-depth research studies conducted among larger groups.

## Figures and Tables

**Table 1:** Distribution of sex in the study group and the control group.

Sex	Study Group (*n* = 41; 100%)	Control Group (*n* = 34; 100%)	Fisher Exact Probability Test
Female	13 (31.7%)	8 (23.5%)	NS (*p* = 0.30)
Male	28 (68.3%)	26 (76.5%)	

**Table 2:** Distribution of age in the study group and the control group.

Age [in Years]	Study Group (*n* = 41; 100%)	Control Group (*n* = 34; 100%)	Chi-Square Test for Association with Yates Continuity Correction
From 3 to 7	18 (43.9%)	16 (47.1%)	NS (*p* = 0.73)
From 7 to 10	13 (31.7%)	8 (23.5%)	
Above 10	10 (24.4%)	10 (29.4%)	

**Table 3:** Types of problems during breastfeeding experienced by the mothers in the study and the control group (question I/3).

Problem	Study Group (*n* = 37; 100%)	Control Group (*n* = 33; 100%)	Fisher Exact Probability Test
Child’s difficulty to hold on to the breast	5 (13.5%)	5 (15.2%)	NS (*p* = 0.56)
Week sucking reflex	4 (10.8%)	1 (3.0%)	NS (*p* = 0.22)
Chaotic sucking (child’s crying, anxiety)	3 (8.1%)	1 (3.0%)	NS (*p* = 0.35)
Child getting tired during breastfeeding	3 (8.1%)	1 (3.0%)	NS (*p* = 0.35)
Short sucking time (child falls asleep)	5 (13.5%)	0	*p* = 0.04
Insufficient/absence of breast milk	7 (18.9%)	6 (18.2%)	NS (*p* = 0.59)
Poor weight gain	3 (8.7%)	2 (6.1%)	NS (*p* = 0.56)
Short lingual frenulum	2 (5.4%)	0	NS (*p* = 0.28)
Regurgitation	6 (16.2%)	2 (6.1%)	NS (*p* = 0.17)
Problem with breast nipples (pain, infection)	4 (10.8%)	4 (12.1%)	NS (*p* = 0.58)
Problem with the mammary gland (infection, abscess)	2 (5.4%)	2 (6.1%)	NS (*p* = 0.65)
Mother’s illness	0	2 (6.1%)	NS (*p* = 0.22)
Child’s illness	1 (2.7%)	2 (6.1%)	NS (*p* = 0.46)
Emotional problem of the mother	0	2 (6.1%)	NS (*p* = 0.22)

**Table 4:** The order in which particular foods are introduced as complementary foods in the study group and the control group (provided: mean; standard deviation; median) (question I/7).

Food	Study Group (*n* = 41)	Control Group (*n* = 34)	Mann-Whitney U Test
Vegetables	1.6; 1.2; 1	1.3; 0.5w; 1	NS (*p* = 0.10)
Fruit	2.0; 1.0; 2	2.1; 1.2; 2	NS (*p* = 0.58)
Meat	3.8; 1.2; 3	3.6; 1.0; 3	NS (*p* = 0.74)
Fish	5.9; 1.6; 6	5.2; 1.6; 5	NS (*p* = 0.14)
Wheat products	4.4; 1.7; 5	4.3; 1.5; 4	NS (*p* = 0.56)
Chicken egg yolk	5.1; 1.7; 6	5.1; 1.1; 5	NS (*p* = 0.64)
Whole chicken egg	6.9; 1.2; 7	7.0; 0.7; 7	NS (*p* = 0.61)
Dairy products—yogurt, Buttermilk	6.3; 1.5; 6	7.4; 1.0; 8	*p* = 0.001

**Table 5:** The necessity of conducting multiple trials in introducing new foods in the study group the and control group (question I/8).

Necessity of Multiple Trials	Study Group (*n* = 41; 100%)	Control Group (*n* = 34; 100%)	Fisher Exact Probability Test
Yes	19 (47.5%)	6 (17.7%)	*p* = 0.006
No	21 (52.5%)	28 (82.3%)	

**Table 6:** What type of complementary food was introduced to child’s died in the study group and the control group (question I/11).

Food Type	Study Group (*n* = 41; 100%)	Control Group (*n* = 34; 100%)	Fisher Exact Probability Test
Ready, store-bought jarred baby food	37 (90.2%)	11 (70.6%)	*p* = 0.03
Food prepared at home and designated solely for the child	37 (90.2%)	29 (85.3%)	NS (*p* = 0.38)
Food at the family table	10 (24.4%)	10 (29.4%)	NS (*p* = 0.41)
Child drinks only milk (breast milk/infant formula)	0	0	---

**Table 7:** Child’s age (in months) at which complementary food with particular texture was introduced to the diet (provided: mean; standard deviation; median) (question I/10).

Food Texture	Study Group (*n* = 41)	Control Group (*n* = 34)	Mann-Whitney U Test
Fluid, mushy	6.2; 5.1; 6.0	5.6; 1.4; 5.5	NS (*p* = 0.78)
Lumpy	9.6; 6.5; 8.0	8.4; 4.2; 7.0	*p* = 0.02
Solid	12.9; 10.1; 9.0	10.5; 4.3; 8.0	*p* = 0.02

**Table 8:** Child’s preferred food texture at the age of 6 months or later in the study group and the control group (question I/18).

Child’s Preferred Food Texture	Study Group (*n* = 41; 100%)	Control Group (*n* = 34; 100%)	Fisher Exact Probability Test
Fluid	15 (36.6%)	9 (26.5%)	NS (*p* = 0.25)
Lumpy	4 (9.8%)	13 (38.2%)	*p* = 0.004
Solid	2 (4.9%)	3 (8.8%)	NS (*p* = 0.41)
Absence of any preference of food texture	24 (58.5%)	15 (44.1%)	NS (*p* = 0.16)

**Table 9:** Accessories used while feeding the child in the study group and the control group (question I/12).

Accessory	Study Group (*n* = 41; 100%)	Control Group (*n* = 34; 100%)	Fisher Exact Probability Test
bottle with a nipple	29 (70.7%)	13 (38.2%)	*p* = 0.005
tea spoon	41 (100%)	33 (97.1%)	NS (*p* = 0.45)
child’s hands	23 (56.1%)	29 (85.3%)	*p* = 0.006
sippy cup	28 (68.3%)	28 (82.4%)	NS (*p* = 0.13)
regular cup	16 (39.0%)	16 (47.1%)	NS (*p* = 0.32)

**Table 10:** Did the feeding require any additional involvement of the caregiver, such as playing with the child, diverting the child’s attention from food? (question I/13).

Additional Involvement	Study Group (*n* = 41; 100%)	Control Group (*n* = 34; 100%)	Fisher Exact Probability Test
Yes	17 (41.5%)	7 (20.6%)	*p* = 0.05
No	24 (58.5%)	27 (79.4%)	

**Table 11:** Did the child watch a cartoon at the time of eating (TV, laptop, tablet)? (question I/14).

Child Watched a Cartoon	Study Group (*n* = 41; 100%)	Control Group (*n* = 34; 100%)	Fisher Exact Probability Test
Yes	13 (31.7%)	5 (14.7%)	NS (*p* = 0.07)
No	28 (68.3%)	29 (85.3%)	

## Supplementary Materials

The following are available online at https://www.mdpi.com/article/10.3390/nu13082687/s1, Questionaire.

## Abbreviations

ICD-10	International Classification of Disease-10,
DSM-5	Diagnostic and Statistical Manual of Mental Disorders-5,
ADOS-2	Autism Diagnostic Observation Schedule, Second Edition,
CDCP	Centers for Disease Control and Prevention,
ASD	Autism Spectrum Disorder.
