# Supplementary material for: Eating Behaviors of Children with Autism—Pilot Study
Source: Nutrients. 2021 Aug 3;13(8):2687. doi: 10.3390/nu13082687 (PMC8398283; doi:10.3390/nu13082687)
Supplement: Supplementary file 1 [file nutrients-13-02687-s001.zip › nutrients-1311499-supplementary.pdf]

Supplementary Material

QUESTIONNAIRE on EATING HABITS

The questionnaire on eating habits has been developed for the purpose of a thorough evaluation of the child's eating habits.
Part I relates to the child's eating habits within the 1st year of life (20 questions).
Part II relates to the child's current eating habits (20 questions). *(unpublished)*
An additional questionnaire, developed by an educational scientist and available in the attachment, relates to the eating habits of the child and its family (12 questions). The majority of the questions are closed-ended questions, to which the respondents will have yes (Y) or no (N) answers to choose from. Some questions are multiple choice questions, to which the respondents will be able to choose and mark the right answer (X). The rest of the questions are open questions, to which the respondents will be able to provide their own descriptive answer.
The tests are anonymous and will be used solely for the purposes related to a research study.
The Researches are available to answer any questions or concerns.

Child personal information:

1. Gender:
2. Date of birth:
3. Date of filling out this questionnaire:

PART I. Child's eating habits in the 1st year of life

1. Was the child breastfed? Y / N
2. Was the mother on any special diet during the breastfeeding period? Y / N

   If the answer is yes, please mark (X) the type of the diet:

a. Dairy-free ( )

b. Lactose-free ( )

c. Gluten-free ( )

d. Egg-free ( )

e. Hypoallergenic ( )

f. Elemental ( )

g. Oligoantigenic ( )

h. Vegetarian ( )

i. Vegan ( )

j. Rotation ( )
k. Candida diet ( )

l. Microbiotic diet ( )

m. Other type ( ), please describe ______________________________

3. Please mark (X) the problems related to breastfeeding:

a. Child's difficulty to hold on to the breast ( )

b. Week sucking reflex ( )

c. Chaotic sucking (child cries, anxiety) ( )

d. Child getting tired during breastfeeding ( )

e. Short sucking time (child falls asleep) ( )

f. Insufficient amount / absence of breast milk ( )

g. Poor weight gain ( )

h. Short lingual frenulum

i. Regurgigation ( )

j. Premature birth ( ), please indicate the pregnancy week ____

k. Problem with breast nipples (pain, infection) ( )

l. Problem with the mammary gland (infection, abscess) ( )

m. Mother's illness ( ), please indicate the type of illness _________________

n. Child's illness ( ), please indicate the type of illness _________________

o. Mother's emotional problems ( )

p. Other ( ), please indicate _____________________________

q. No problems with breastfeeding ( )

4. Was the child fed with infant formula (otherwise referred to as artificial milk, milk mixture, powder milk)? Y / N

   If the answer is yes, please indicate the duration: starting at ____ month, until ____

5. Was the child fed with a special infant formula Y / N

If the answer is yes, please mark (X) the type of the special infant formula used:

a. Pre, Nenatal (milk substitute, for prematurely born and with low birth weight infants) ( )

b. HA (hypoallergenic, low degree of hydrolysis of protein hydrolysates) ( )

c. Nutramigen (high degree of hydrolysis of casein hydrolysates) ( )

d. Pepti (high degree of hydrolysis of whey protein hydrolysates ( )

e. Neocate, Bebilon Amino (elemental formula) ( )

f. AR (antireflux, antiregurgigation) ( )

g. R/GR (with rice starch) ( )

h. SL/0-lac (soy, lactose-free) ( )

i. Comfort/AntiColic (infant formula for colic) ( )

j. Sensitive (for infants with sensitive digestive system) ( )

k. HN (to treat diarrhea) ( )

6. When were the first complementary foods introduced in the child's diet (i.e. other than the mother's milk or the infant formula) ____ (please indicate the month of the child's life)

7. Please indicate the order in which the following complementary foods were introduced by marking them with numbers from (1) to (8)

a. Vegetables ( )

b. Fruit ( )

c. Meat ( )

d. Fish ( )

e. Cereal products ( )

f. Chicken egg yolk ( )

g. Whole chicken egg ( )

h. Dairy products- yogurt, kefir ( )

8. Was there a need to attempt to introduce certain complementary foods several times during the time of broadening the child's diet? Y / N

9. Please mark (X) any foods that were not tolerated by the child:

a. Cow's milk ( )

b. Chicken egg ( )

c. Wheat products ( )

d. Soy ( )

e. Fish ( )

f. Nuts ( )

g. Chocolate ( )

10. Please provide the child's age (in months) when the complementary foods with the particular texture were introduced in the child's diet:

a. Fluid, mushy ____ (month)

b. Lumpy ____ (month)

c. Solid ____ (month)

11. Please mark (X) the types of complementary foods that were introduced to the child's diet:

a. Ready, store-bought jarred baby food ( )

b. Food prepared at home and designated solely for the child ( )

c. Food at the family table ( )

d. Child drinks only milk (breast milk / infant formula) ( )

12. Please mark the accessories used while feeding the child:

a. Bottle with a nipple ( )

b. Spoon ( )

c. Child's hands ( )

d. Sippy cup ( )

e. Regular cup ( )

13. Did the feeding require any additional involvement of the caregiver, such as playing with the child, diverting the child's attention from food? Y / N

14. Did the child watch a cartoon at the time of eating? (TV, laptop, tablet) Y / N

15. Was the child breastfed during the sleep? Y / N

16. Was the child fed during a family meal time with other family members present? Y / N

17. Please mark (X) any of the following words to describe the child's appetite during the 1st year of life:

a. Normal ( )

b. Increased ( )

c. Decreased ( )

d. Lack of appetite ( )

e. Selective ( ), please describe the type of foods preferred by the child ________________

18. Please mark (X) the texture of the foods preferred by the child at the age above 6 months of life :

a. Fluid ( )

b. Lumpy ( )

c. Solid ( )

d. Lack of any preference of the type of food texture ( )

19. Please mark (X) the type of food texture not tolerated by the child at the age above 6 months of life:

a. Fluid ( )

b. Lumpy ( )

c. Solid ( )

d. Lack of any intolerance of the type of food texture ( )

20. Did the child require any restrictive diets in the 1st year of life? Y / N
If the answer is yes, please mark (X) the type of the restrictive diet:

a. Dairy-free ( )

b. Lactose-free ( )

c. Gluten-free ( )

d. Egg-free ( )

e. Hypoallergenic ( )

f. Elemental ( )

g. Oligoantigenic ( )

h. Vegetarian ( )

i. Vegan ( )

j. Rotation ( )

k. Candida diet ( )

l. Microbiotic diet ( )

m. Other type ( ), please describe ______________________________
